# Development of mild encephalitis with a reversible splenial lesion prior to the diagnosis of Kawasaki disease: a pediatric case report

**DOI:** 10.3389/fped.2025.1652101

**Published:** 2025-08-13

**Authors:** Hirokazu Yamanouchi, Sagano Onoyama, Kentaro Marutani, Nobutaka Harada, Yuji Ueno, Hikaru Kanemasa, Ryutaro Kira, Shuya Kaneko, Masaki Shimizu, Takayuki Hoshina

**Affiliations:** ^1^ Kawasaki Disease Center, Fukuoka Children’s Hospital, Fukuoka, Japan; ^2^ Department of Pediatric Neurology, Fukuoka Children’s Hospital, Fukuoka, Japan; ^3^ Department of Pediatric Infectious Diseases and Immunology, Fukuoka Children’s Hospital, Fukuoka, Japan; ^4^ Department of Pediatrics and Developmental Biology, Institute of Science Tokyo, Tokyo, Japan; ^5^ Department of Pediatrics, Perinatal and Maternal Medicine, Graduate School of Medical and Dental Sciences, Institute of Science Tokyo, Tokyo, Japan; ^6^ General Medical Department, Fukuoka Children’s Hospital, Fukuoka, Japan

**Keywords:** Kawasaki disease, mild encephalitis with a reversible splenial lesion, coronary artery abnormality, intravenous immunoglobulin, case report, presenting symptoms

## Abstract

Kawasaki disease (KD) rarely causes neurological complications. KD is diagnosed based on symptoms alone and can be very difficult to diagnose if other symptoms appear in febrile children before the main symptoms of KD. A 5-year-old boy with fever and consciousness disturbance was hospitalized and diagnosed with mild encephalitis/encephalopathy with reversible splenial lesion (MERS). The fever and consciousness disturbance resolved with intravenous methylprednisolone for 3 days (30 mg/kg/day) and intravenous immunoglobulin (IVIG; 1 g/kg/day) for 2 days, which was initiated as treatment for MERS. However, bilateral conjunctival injections, redness of the lips, and membranous desquamation of the fingers were observed, followed by recurrence of fever four days after the initial treatment. Echocardiography revealed dilation of the right coronary artery (RCA). The patient was diagnosed with incomplete KD and was treated with high-dose IVIG and oral aspirin based on the presence of four major KD symptoms and coronary artery dilation. After treatment, he showed defervescence, and the RCA showed no further dilation on echocardiography. Clinicians should recognize that the development of MERS may precede the diagnosis of KD in some patients. In addition, patients with MERS of unknown etiology, leukocytosis, and elevated serum CRP levels should be closely monitored because of the possibility of KD.

## Introduction

Kawasaki disease (KD) is a systemic vasculitis of unknown origin that predominantly affects children under five years of age (1). KD is commonly complicated by cardiovascular diseases, including coronary artery abnormalities (CAAs), but it can also cause complications in other organs (2). Neurological complications associated with KD are relatively rare, with encephalopathy occurring in only 0.09% of patients (3). KD is diagnosed on the basis of symptoms alone, which can be very difficult to diagnose if other symptoms appear in febrile children before the main symptoms of KD (e.g., rash, red eyes, strawberry tongue, enlarged lymph nodes in the neck, and swelling of the palms and soles) (2).

We herein report the case of a pediatric patient who was hospitalized for fever and consciousness disturbance caused by mild encephalitis/encephalopathy with a reversible splenial lesion (MERS), characterized by a reversible lesion with homogenously reduced diffusion in the corpus callosum, which recovered almost completely within one month (4). Symptoms of KD emerged while the patient was receiving treatment for the MERS. It is clinically important to keep in mind the existence of KD, potentially presenting with MERS-like features, before the classic diagnostic criteria are met. In addition, we reviewed the characteristics of patients who developed MERS before the diagnosis of KD, including previously reported cases.

## Case report

A previously healthy 5-year-old boy was admitted to our hospital for evaluation and treatment of consciousness disturbance and fever. The patient had a fever, headache, abdominal pain, and vomiting on the day before admission. On the morning of admission, he spoke incoherently and found it difficult to make eye contact. The patient was referred to our hospital.

**Figure 1:**
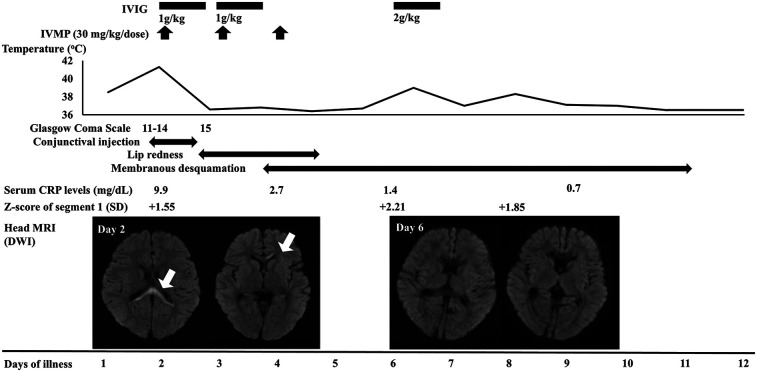
Clinical course and the serial changes of laboratory and imaging findings. Segment 1 indicates the right coronary artery. The diameter was measured by echocardiography. IVIG, intravenous immunoglobulin; IVMP, intravenous methylprednisolone; CRP, C-reactive protein; SD, standard deviation; MRI, magnetic resonance imaging; DWI, diffusion-weighted imaging.

On admission, the patient exhibited an altered level of consciousness, with a Glasgow Coma Scale score of 11–15 (E4V3-5M4-6). His body temperature, heart rate, and blood pressure were 40.3°C, 160 bpm, and 88/62 mmHg, respectively. He had bilateral conjunctival injections. Nuchal rigidity and Kernig's sign were positive. Laboratory findings showed a peripheral white blood cell (WBC) count of 24.02 × 10^9^/L with 87.2% neutrophils, a platelet count of 197 × 10^3^/μl, a serum C-reactive protein (CRP) level of 9.92 mg/dl, aspartate aminotransferase (AST) level of 37 U/L, serum procalcitonin level of 27.6 ng/ml and serum sodium level of 130 mEq/L. A cerebrospinal fluid (CSF) analysis showed mild pleocytosis (mononuclear cells, 10 µg/µl), with normal protein and glucose levels. Magnetic resonance imaging (MRI) of the head at admission revealed high-intensity areas in the splenium and genu of the corpus callosum, whereas the apparent diffusion coefficient map revealed low-intensity areas in the same lesions (Figure 1). Although KD was suspected because of fever, conjunctival injection, leukocytosis, and elevated serum CRP levels, echocardiography revealed no abnormalities in the coronary arteries.

On the first day of admission (the second day of illness), intravenous methylprednisolone for 3 days (30 mg/kg/day) and intravenous immunoglobulin (IVIG) (1 g/kg/day) for 2 days were initiated as treatment for acute encephalitis (Figure 1). The fever resolved and the patient's consciousness improved on the third day of illness. The abnormal findings were found to have resolved on head MRI performed on the seventh day of illness. Based on the head MRI findings and the clinical course, the patient was diagnosed with MERS.

On the fourth and fifth days of illness, the patient had lip redness and membranous desquamation of the fingers, respectively, and had fever again on the seventh day of illness. Echocardiography revealed dilation of the right coronary artery. Based on the presence of four major KD symptoms and coronary artery dilation, the patient was diagnosed with incomplete KD and treated with high-dose IVIG (2 g/kg) and oral aspirin (30 mg/kg/day). After treatment, he had defervescence, and the coronary artery showed no further dilation on echocardiography. He was discharged on the 13th day of illness without any neurological or cardiac sequelae.

Serum concentrations of proinflammatory cytokines, including interleukin (IL)-6, IL-18, soluble tumor necrosis factor receptor type II (sTNF-RII), and C-X-C motif chemokine ligand 9 (CXCL9), were measured using enzyme-linked immunosorbent assay kits in the sample collected at admission, as previously reported (5). The serum levels of IL-6, IL-18, sTNF-RII, and CXCL9 were 150 pg/ml [reference range (r.r.) < 3 pg/ml], 1,015 pg/ml (r.r. < 500 pg/ml), 10,367 pg/ml (r.r. 829–2,262 pg/ml), and 2,886 pg/ml (r.r. 31–83 pg/ml), respectively. Relative to the serum IL-18 levels, the serum levels of IL-6, sTNF-RII, and CXCL9 were more elevated, and among them, the increase in serum IL-6 levels was particularly notable, which is similar with the pattern observed in KD (5).

## Discussion and review of the relevant literature

KD can affect various organ systems and may occasionally present unusual clinical features (2). Some patients are diagnosed with and treated for acute appendicitis, pneumonia, and encephalitis/encephalopathy before being diagnosed with KD (6–9). The patient was also diagnosed with and treated for MERS prior to the diagnosis of KD. We conducted a PubMed search and reviewed reports presenting the characteristics of patients who developed MERS prior to the diagnosis of KD. A total of five patients have been reported previously (Table 1) (8–11). When the analysis included the present case, the proportion of female patients (67%) and the median age (7 years) were higher than those in a recent nationwide survey of all KD patients in Japan (12). Previous surveys in Japan have shown that the proportion of female patients with MERS was not high, although the number of patients was relatively small (4). It is unclear why there was a female predominance in the six patients presented. The median interval between the diagnosis of MERS and KD was 3 days (1–11 days). Three patients (50%) had CAA complications. None of the patients had any neurological sequelae. In patients with other diseases prior to the diagnosis of KD, the incidence of CAA is considered relatively high, possibly due to the delayed diagnosis of KD (2, 6, 7). In addition, extended inflammatory activity is a risk factor for complications (8). Of the six patients with MERS, three who developed CAA had a delay before being diagnosed with KD (Patient 1) or were refractory to initial treatment for KD (Patients 3 and 6) (Table 1). Clinicians should be aware that the development of MERS may precede the diagnosis of KD in some patients, and patients with an unknown MERS etiology, leukocytosis, and elevated serum CRP levels should be closely observed due to the possibility of KD.

**Table 1:** The characteristics of the 6 patients developing MERS prior to the diagnosis of KD.

Pt.	Age (years)	Sex	Days of Dx. of KD	Days of Dx. of MERS	Treatments for MERS	Treatments after Dx. of KD	Neurological sequelae	CAAs	Ref.
1	14	F	17	6	IVIG (400 mg/kg/dose)	IVIG (1.4 g/kg/dose)	−	+	8
2	7	F	5	3	None	IVIG (2 g/kg/dose), ASA	−	−	9
3	8	M	5	1	None	IVIG (2 g/kg/dose, 3 times) ASA, CyA, IFX	−	+	10
4	7	F	5	3	None	IVIG (2 g/kg/dose), ASA	−	−	10
5	2	F	3	2	None	IVIG (2 g/kg/dose, twice) ASA, IVMP, IFX	−	−	11
6	5	M	7	2	IVIG (1 g/kg/dose, twice) IVMP	IVIG (2 g/kg/dose), ASA	−	+^a^	Ours

MERS, mild encephalitis/encephalopathy with a reversible splenial lesion; KD, Kawasaki disease; CAA, coronary artery abnormality; IVIG, intravenous immunoglobulin; ASA, aspirin; CyA, cyclosporin A; IFX, infliximab; IVMP, intravenous methylprednisolone; Pt., patient; Dx., diagnosis; Ref., reference;

^a^ Transient dilation.

MERS is a condition characterized by fever, abnormal behavior, disturbance of consciousness, and seizures, primarily occurring post-infection (4). The pathogenesis of MERS remains unclear; however, several mechanisms have been proposed, including post-infectious cytokine storms, intramyelinic edema caused by systemic inflammatory responses, brain edema associated with hyponatremia, and transient local infiltration of inflammatory cells (4). In patients with KD, retropharyngeal edema is often found and misdiagnosed as a retropharyngeal abscess (2). In addition, the histopathological findings of the appendix in patients with KD, primarily diagnosed with acute appendicitis, showed inflammatory changes with edema (6). As patients with KD are prone to developing edema in various parts of the body, MERS should also be recognized as a complication. In the present case, hyponatremia (130 mEq/L) in the acute phase of KD may have contributed to the onset of MERS. Further studies are needed to determine the mechanism underlying the development of MERS in KD.

There have been several reports of cytokine profile analyses of the serum or CSF of patients with KD or MERS. Kaneko et al. compared the cytokine profiles of patients with KD and KD-like diseases and reported that serum IL-6 levels were particularly elevated in those with KD shock syndrome, a more severe form of KD with a high incidence of CAAs (5). In patients with acute focal bacterial nephritis-associated MERS, various inflammatory cytokines (e.g., IL-6, IL-10, CXCL10, TNF-α, and interferon-γ) were elevated in both serum and CSF during the acute phase of the disease and these cytokines returned to normal levels after two weeks (13). Another study showed that the CSF IL-6 levels were elevated in patients with virus-associated MERS, but the levels were not as high as those in patients with KD or acute focal bacterial nephritis (14). In a previous report, inflammatory cytokines were measured in only one patient with KD complicated by MERS, and, as in our patient, the serum IL-6 level was high, but the serum IL-18 level was not (15). Taken together, although IL-6 is elevated even in MERS associated with other diseases and cannot be considered specific to KD, a detailed assessment of its elevation or the measurements of inflammatory cytokines other than IL-6, including IL-18, sTNF-RII, and CXCL9, may be helpful in the diagnosis of KD.

In conclusion, MERS should be considered as a complication of KD. In addition, neurological symptoms caused by MERS may appear as the initial symptoms of KD. Clinicians should maintain a high level of suspicion for KD in children presenting with MERS-like neurological features and elevated inflammatory markers, particularly if classic KD signs emerge later. Further large-scale studies are needed to determine the mechanism underlying the development of MERS in KD and the epidemiology of patients with these two diseases, including the sex ratio.

## Data Availability

The original contributions presented in the study are included in the article/Supplementary Material, further inquiries can be directed to the corresponding author.
